# The Role of Marital Status in Sexual Violence Against Women Among Ghanaian Couples: Evidence From the 2022 Ghana Demographic and Health Survey

**DOI:** 10.34172/jrhs.9086

**Published:** 2025-09-15

**Authors:** Leny Latifah, Agung Dwi Laksono, Marizka Khairunnisa, Diah Yunitawati, Sri Handayani

**Affiliations:** ^1^National Research and Innovation Agency, Republic of Indonesia, Jakarta, Indonesia; ^2^Persakmi Institute, Surabaya, Indonesia

**Keywords:** Cohabitation, Marital status, Sexual violence, Violence, Women, Couple

## Abstract

**Background:**

Cohabitation is vulnerable from a legal perspective. However, legal marriage does not exempt women from experiencing sexual abuse, while marriage is often misused as a justification. This study aimed to examine the impact of marital status on the incidence of sexual violence against women within couples.

**Study Design:**

A cross-sectional study.

**Methods:**

The secondary analysis examined the 2022 Ghana Demographic and Health Survey, which included data from 8811 respondents. The study used sexual violence and marital status as outcome and exposure variables, respectively. Meanwhile, five control variables, including age, marital status, education, employment, wealth, and recent sexual activity, were analyzed in this study. Finally, the results were analyzed by binary logistic regression (*P*<0.05) using SPSS 21.

**Results:**

Sexual violence was reported by 5.3% of women living with a partner and 2.6% of married women (*P*<0.001). Based on marital status, women in a cohabitation relationship were 1.57 times more likely to experience sexual violence than married ones (adjusted odds ratio [AOR]: 1.857, 95% confidence interval: 1.857–1.858). Several factors were also significantly associated with increased risk of sexual violence, including urban residence (AOR: 1.139), younger age (e.g., 20–24 years: AOR: 1.766), lower education/no education (AOR: 2.045), unemployment (AOR: 1.415), lack of pregnancy (AOR: 1.221), recent sexual activity (AOR: 1.266), and women in middle-income and richer groups (AOR: 1.175 and AOR: 1.414).

**Conclusion:**

The evaluation revealed that marital status was related to sexual violence against women among Ghanaian couples. Women living in cohabitation with a partner were more likely to experience sexual violence than married women.

## Background

 Sexual violence is any act or effort to engage in sexual activities without consent.^1^ It is characterized by coercing an individual into sexual intercourse without their consent, engaging in sexual relations due to having a fear of their partner, and/or being compelled to commit acts deemed humiliating.^2^

 In addition, sexual violence harms victims physically, psychologically, neurocognitively, and socially, and even leads to death.^3-6^ Women who have experienced sexual violence exhibit a significantly elevated incidence of anxiety disorders, depression, post-traumatic stress disorder, and substance abuse. Post-traumatic stress disorder is among the most clinically significant health consequences of domestic violence.^4,5^ Further, sexual violence is associated with an increased risk of disease, including cardiovascular disease and type 2 diabetes.^4^

 It is noteworthy that sexual violence is a worldwide public health and human rights issue. According to the World Health Organization, about 27% of women aged 15–49 years are estimated to have experienced physical or sexual violence from a partner in their lifetime.^7^ In Sub-Saharan Africa, the prevalence of women reporting such experiences is higher (around 33%).^7^ Meanwhile, in Ghana, based on data from the Ghana Demographic and Health Survey (GDHS) report in 2022, 36% of women aged 15–49 years experienced various forms of intimate partner violence (IPV), which includes emotional, physical, and sexual violence, and 14% of them reported experiencing sexual violence by any perpetrator.^8^

 Research consistently indicates that several factors influence the prevalence of intimate sexual violence, including education and socioeconomic status. Women with lower levels of education and income are more susceptible to experiencing sexual violence from their partners.^2^ This disparity is deeply rooted in patriarchal culture, which perpetuates power imbalances in relationships and contributes to IPV, including physical and sexual aggression.^9^ Furthermore, personal factors, such as exposure to violence in childhood, can heighten a man’s likelihood of perpetrating IPV. Women whose partners struggle with substance abuse, particularly alcohol, are also at an increased risk of encountering sexual violence from their partners.^10^

 For women, legal marital status is more substantial in law than cohabitation status. Cohabitation refers to the relationship between a man and a woman living together, regardless of whether they have children. Cohabitation is not acknowledged by statutory or customary law, unlike marriage under state law.^11^ Women in cohabiting relationships usually have less bargaining power due to limitations of social and financial status.^12^ Lacking legal protection leads women to be at risk of property ownership losses^11^ and vulnerable to IPV.^12^

 However, legal marriage does not ensure that women are exempt from experiencing sexual abuse. Marriage is often misused as a justification for sexual abuse. In the Bangladeshi legal system, marital rape is not classified as an act of sexual assault, as the husband perpetrates it in the marriage.^13^ Additionally, the law has challenges in intervening, as victims of abuse are often afraid of filing legal complaints, and domestic violence is perceived as a private matter.^2^ Considering the context, the objective of the study is to examine the impact of marital status on the incidence of sexual violence against women within Ghanaian couples.

## Methods

###  Study design and data source

 This secondary analysis used the 2022 GDHS, a cross-sectional survey conducted by the Ghana Statistical Service. The 2022 GDHS utilized a questionnaire adapted to DHS-8 standards, and the required data were collected using the Computer-Assisted Personal Interviewing system.^8^

 Women aged 15–49 were investigated in this study. The 2022 GDHS used stratification and multistage random sampling. The response rate of the survey was 98% for women. Based on the sampling methods, 7,652 women were found to live in rural areas.

###  Setting

 The study was conducted in rural Ghana nationally. The urban-rural classification refers to the Ghana Statistical Service. The fieldwork for the survey was performed from October 17, 2022, to January 14, 2023.^14^

###  Ethical considerations

 The GDHS data collection was performed in accordance with the ethical guidelines established by the Demographic and Health Survey (DHS) Program. The GDHS ensured the anonymization of the identities of all participants within the dataset. Participants submitted consent forms for this study, while parents or custodians provided consent for participants who were minors (under the age of 16). Participation was voluntary, with strict confidentiality maintained throughout the study. Field data collectors received specialized training to handle sensitive questions responsibly and respectfully. Interviews were conducted with measures to ensure privacy, minimizing potential distress or harm to participants.^15^

###  Outcome variable

 Sexual violence was the outcome variable in the study. It entails the physical coercion to engage in sexual intercourse against your will, the physical compulsion to do unwanted sexual activities, or the use of threats or other means to compel you to undertake sexual actions you do not consent to.^8^ Sexual violence comprises No and Yes.

###  Exposure variables

 The study’s exposure variable was marital status. The survey identified two types of marital status, namely, married and living with a partner.

###  Control variables

 Eight control variables were examined in this study, including residence type, age group, education level, employment status, wealth status, parity, expectant status, and recent sexual activity. Urban and rural areas were included in the classification of residence. The participants were categorized into seven groups in terms of age (15–19 years, 20–24 years, 25–29 years, 30–34 years, 35–39 years, 40–44 years, and 45–49 years). Moreover, they were divided into unemployed and employed groups.

 The wealth quintile of the products owned by a household was employed in the study to evaluate the household’s economic status. The survey assessed the quantity and classification of objects that families maintained within their households. The research examined various factors to project revenue, including televisions, bicycles, automobiles, and demographic variables. The components of the primary structure, the restroom facilities, and the potable water supply system underwent analysis. Principal component analysis was utilized in the investigation to calculate the score. It should be noted that 20% of the population was surveyed, and the household scores of each participant were compiled to produce quintiles representing the nation’s wealth distribution. Subsequently, five categories were delineated through further subdivision of the quintiles. Wealth status was categorized into five quintiles, representing the poorest (quintile 1), poorer (quintile 2), middle (quintile 3), richer (quintile 4), and richest (quintile 5) groups.^15^

 The study separated parity into primiparous (0–1 child), multiparous (2–4 children), and grand multiparous ( > four children) groups. In the interim, being expectant was divided into “No” and “Yes”. Furthermore, recent sexual activity included never having sex, being active in the last four weeks, and not being active in the previous four weeks.

###  Data analysis

 The study initially employed the chi-square test. Subsequently, a collinearity test was conducted to determine whether there was a statistically significant relationship among the independent variables. A binary logistic regression analysis was performed, with a significance level set at *P*< 0.05. Additionally, the International Business Machines Statistical Package for the Social Sciences (IBM SPSS) Statistics 21 software was utilized to conduct the statistical analyses for the survey.

## Results

 The 2022 DHS in Ghana achieved a high response rate. The survey achieved a response rate of 99% and 98% for households and women aged 15–49, respectively.^14^ The investigation revealed that the incidence of sexual assault against women among Ghanaian couples was approximately 7.6%. Nearly 78.1% of Ghanaian couples were married, while the remaining 21.9% were cohabiting with a partner. Table 1 presents the findings of the bivariate evaluation.

**Table 1:** The descriptive statistics of marital status and other variables (N = 8811)

**Variables**	**Married (n=6884)**		**Living With a Partner (n=1927)**		
	**Number**	**Percent**	**Number**	**Percent**	***P *** **value**
Experienced sexual violence					
No	6,705	97.4	6,519	94.7	0.001
Yes	179	2.6	365	5.3	
Residence					
Urban	3,635	52.8	3,531	51.3	0.001
Rural	3,249	47.2	3,353	48.7	
Age group (year)					
15–19	89	1.3	351	5.1	0.001
20–24	558	8.1	1,528	22.2	
25–29	1,115	16.2	1,570	22.8	
30–34	1,556	22.6	1,191	17.3	
35–39	1,459	21.2	1,115	16.2	
40–44	1,232	17.9	695	10.1	
45–49	874	12.7	441	6.4	
Education level					
No education	2,010	29.2	936	13.6	0.001
Primary	943	13.7	1,280	18.6	
Secondary	3,125	45.4	4,420	64.2	
Higher	805	11.7	248	3.6	
Employment status					
Unemployed	888	12.9	1,143	16.6	0.001
Employed	5,996	87.1	5,741	83.4	
Wealth status	6,884				
Poorest	1,570	22.8	833	12.1	0.001
Poorer	1,101	16.0	1,666	24.2	
Middle	1,157	16.8	1,735	25.2	
Richer	1,335	19.4	1,914	27.8	
Richest	1,721	25.0	737	10.7	
Pregnant status					
No	6,175	89.7	6,079	88.3	0.001
Yes	709	10.3	805	11.7	
Parity					
Primiparous	1,370	19.9	2,471	35.9	0.001
Multiparous	3,566	51.8	3,242	47.1	
Grand multiparous	1,948	28.3	1,170	17.0	
Recent sexual activity					
Active in the last 4 weeks	4,481	65.1	4,516	65.6	0.001
Not active in the last 4 weeks	2,403	34.9	2,368	34.4	

 Based on experienced sexual violence, women who lived with a partner were in a higher proportion than married women (Table 1). Meanwhile, according to the residence type, urban areas had a higher ratio than rural areas in terms of living with a partner. In terms of age group, women aged 25–29 had the highest ratio of living with a partner.

 Regarding the education level, women with secondary education had the highest ratio of living with a partner (Table 1). Based on employment status, employed women dominated both kinds of marital status. Furthermore, more prosperous women had the highest proportion in the cohabitation group.

 Concerning pregnant status, women who were not pregnant dominated the two groups of marital status (Table 1). According to parity, multiparous women had the highest ratio of cohabitation kind. Based on recent sexual activity, women who were active in the last four weeks were almost two times more likely than those who were not active during the previous four weeks in the cohabitation group.

 The research subsequently examined collinearity. The experimental results indicated that the independent variables exhibited no collinearity. The variance inflation factor values for each variable were concurrently below 10.00, per the statistics. Further, the average tolerance values for all variables exceeded 0.10. The multicollinearity test demonstrated no substantial connection among two or more independent variables, which informed assessments of the regression model.

Table 2 provides the findings of the binary logistic regression results of sexual violence. Based on marital status, Ghanaian women who lived with a partner were 1.57 times more likely to experience sexual violence than those who were married (adjusted odds ratio [AOR]: 1.857, 95% confidence interval [CI]: 1.857–1.858). The results showed that eight control variables were related to sexual violence against women among Ghanaian couples.

**Table 2:** The binary logistic regression results of sexual violence (N = 8811)

**Predictors**	**Adjusted OR (95% CI)**	***P*** ** value**
Marital status		
Married	Ref.	
Living with a partner	1.857 (1.857–1.858)	0.001
Residence	-	-
Urban	1.139 (1.138–1.139)	0.001
Rural	Ref.	
Age group (year)		
15-19	0.702 (0.701–0.703)	0.001
20-24	1.766 (1.765–1.767)	0.001
25-29	1.780 (1.779–1.781)	0.001
30-34	1.500 (1.499–1.501)	0.001
35-39	1.871 (1.870–1.872)	0.001
40-44	1.502 (1.501–1.503)	0.001
45-49	Ref.	
Education level		
No education	2.045 (2.044–2.047)	0.001
Primary	1.722 (1.721–1.723)	0.001
Secondary	1.524 (1.523–1.525)	0.001
Higher	Ref.	
Employment status		
Unemployed	1.415 (1.415–1.416)	0.001
Employed	Ref.	
Wealth status		
Poorest	0.760 (0.759–0.760)	0.001
Poorer	0.879 (0.879–0.880)	0.001
Middle	1.175 (1.175–1.176)	0.001
Richer	1.414 (1.414–1.415)	0.001
Riches	Ref.	
Currently pregnant		
No	1.221 (1.221–1.222)	0.001
Yes	Ref.	
Recent sexual activity		
Active in the last 4 weeks	1.266 (1.266–1.267)	0.001
Not active in the last 4 weeks	Ref.	
Parity		
Primiparous	0.488 (0.488–0.489)	0.001
Multiparous	0.612 (0.612–0.613)	0.001
Grand multiparous	Ref.	

*Note*. OR: Odds ratio; CI: Confidence interval.

 Regarding the residence type, women in urban areas were 1.139 times more likely than those in rural areas to be sexually violent in Ghana (CI: 1.138–1.139, Table 2). In addition, four demographic characteristics were associated with sexual violence, including age group, education level, employment status, and wealth status.

 As regards current pregnancy status, women who were not pregnant were 1.221 times more likely to experience sexual violence from their partner than those pregnant (AOR: 1.221, 95% CI: 1.221–1.222, Table 2). Based on recent sexual activity, women who were active in the last four weeks were 1.266 times more likely than those not active in the previous four weeks to be sexually violent (AOR: 1.266, 95% CI: 1.266–1.267). Furthermore, according to parity, all parity types were more likely than grand multiparous women to experience sexual violence from their partner.

## Discussion

 The study results confirmed the existence of sexual violence against women among Ghanaian couples. The risks of sexual violence against women may be attributed to societal norms that emphasize spousal rights to sex, potentially leading to coercion. Power imbalances within relationships and economic dependency could further increase their vulnerability. These findings highlight the need for policies and interventions that promote consent and protect women’s rights within intimate relationships.^2^

 Moreover, women in cohabitation relationships were more likely to experience sexual violence than married women. Cohabiting women may be because they have less formal protection, their relationships might be less stable, or society views living together differently than marriage. Not having formal marriage vows can lead to unequal power between partners and make one partner more open to pressure or manipulation. These results revealed that we need specific actions to reduce IPV among couples who live together.^2,16^ The results of this nationally representative study are in line with those of qualitative research on female students at Ghanaian universities. Our findings demonstrated that cohabiting individuals frequently encounter pressures from society to either formalize their unions through marriage or terminate the ties. The relationships are marked by IPV and substandard relationship quality, with women experiencing these consequences with greater intensity than men.^17^

 Regarding the residence type, women in urban areas were more likely to be sexually violent in Ghana than those in rural areas. It is possibly due to increased reporting, different social norms, or greater exposure to risk factors associated with urban living.^18^ National data from Indonesia showed similar results, with physical and/or sexual violence more common among women in urban areas.^19^ In contrast, different results found in a population-based analysis in Turkey indicated that women living in urban areas were less likely to experience sexual violence, due to better information access and higher awareness of rights and laws in urban women.^2^ These differences suggest that the relationship between the incidence of sexual violence and geographical location is highly variable and influenced by other factors.

 Based on the results, four demographic characteristics were associated with sexual violence, including age group, education level, employment status, and wealth status. Younger women may be more vulnerable due to power imbalances in relationships, while lower education levels could limit awareness of rights and access to support services. Unemployed women or those with lower economic status may face increased dependency on their partners, making them more susceptible to coercion and abuse.^20-22^

 Conversely, women with higher wealth status might have greater autonomy and resources to resist or report violence, potentially reducing their risk. Employment can provide financial independence, empowering women to leave abusive situations or seek help. These results highlight the need for economic empowerment, educational initiatives, and legal protections to reduce women’s vulnerability to sexual violence.^23,24^

 Based on recent sexual activity, women who were active in the last four weeks were more likely to be sexually violent than those who were not active in the previous four weeks. It possibly indicates coercion within relationships or power imbalances influencing consent. This research showed a higher proportion of young women in cohabitation relationships. It also exhibited lower levels of employment among cohabiting women. Young women who are at their peak sexual activity may have increased exposure to potential perpetrators. Young people, especially those with lower levels of education, typically have more partners, have less sexual knowledge, and are less able to refuse unwanted sex, thereby increasing the risk of unwanted sex. Several factors likely contribute to this vulnerability.^25^ However, these causal relationships are not always transparent and require further research.

 Regarding current pregnancy status, non-pregnant women were more likely to experience sexual violence from their partner than those who were pregnant. Non-pregnant women faced a higher risk of sexual violence from their partners, possibly due to changes in relationship dynamics, reduced perceived vulnerability, or differing partner attitudes toward pregnancy. The findings of this study align with those of a similar survey conducted in Hong Kong, demonstrating a decrease in sexual violence from partners during pregnancy. Family support and partner involvement may serve as protective factors.^26^ A study in Iran highlighted significant differences in social support between pregnant and non-pregnant women, particularly regarding support from spouses and friends. Pregnant women reported receiving excellent support during pregnancy.^27^ Similarly, research in Bangladesh indicated a reduction in sexual violence during pregnancy compared to before pregnancy. The vulnerability of pregnant women may lead them to leverage their condition as a strategy to avoid sexual violence from their spouses.^28^ Additionally, pregnant women are less likely to experience sexual violence from their partners due to reduced sexual activity during pregnancy. Research conducted in Poland revealed that pregnancy can cause changes in sexual desire and function, influenced by physical, emotional, and hormonal shifts. One of the main reasons couples avoid sex during pregnancy is concern for the health of the baby.^29^

 Furthermore, according to parity, all parity types were more likely to experience sexual violence from their partners than the grand multiparous type. Women who have fewer children are at a greater risk of experiencing sexual abuse from their partners than those who have four or more children. The higher risk might be due to differences in power in the relationship, cultural beliefs, or the relationship’s stability.^22^ This finding, while not identical, conforms to a Japanese study, implying that multiparous women are more susceptible to IPV than primiparous women.^30^ Conversely, research undertaken in East Africa suggests that the incidence of partner sexual violence is higher among women with several children than among nulliparous women.^31^

 The study’s strength lies in its utilization of extensive data for research, enabling it to represent Ghana nationally. Conversely, utilizing secondary data restricts the study in analyzing a limited number of factors supplied by the survey owner. This study could not explore some variables substantially associated with sexual assault, including spouse traits, cohabitation with in-laws, and parity.^32,24^ The other limitation of this study was the question about sexual violence formed as a yes or no question. Therefore, the answer may not capture the complexity of experiences, and respondents may not have felt comfortable disclosing truthful information. The methods of answering the question were self-reported; hence, self-reports on sensitive topics like sexual violence may be subject to underreporting or social desirability bias. However, the DHS survey instrument was adapted from validated tools and designed to minimize bias.

**Highlights**

- This was nationally representative research among couples in Ghana.
- Among Ghanaian couples, 78.1% were married, while 21.9% were cohabiting.
- Marital status was associated with intimate sexual violence against women among couples.
- Women in cohabitation relationships were more likely to experience sexual violence.

## Conclusion

 Based on the findings, the evaluation confirmed that marital status was related to sexual violence against women among Ghanaian couples. Women who lived with a partner were more likely to experience sexual violence than married women. It is imperative to enhance legal protections and support systems for women in cohabiting relationships, thereby ensuring their equitable access to resources for the prevention and resolution of sexual violence. Public awareness campaigns and community-based interventions should advocate for healthy relationship dynamics, underscoring the principles of consent and mutual respect. Additionally, policymakers should consider integrating targeted programs into existing domestic violence prevention initiatives to support at-risk women, particularly those in cohabiting unions.

## Competing Interests

 The authors have declared that there is no conflict of interests.

## Ethical Approval

 The study utilized secondary data from the 2022 GDHS for materials analysis. The author secured permission to use data from the website https://dhsprogram.com for this study.

 The 2022 GDHS adheres to the standard DHS methodology as delineated by The DHS Program (DHS-7). This methodology has received endorsement from the Ghana Health Service Ethics Review Committee and the Institutional Review Board of ICF International, following an initial assessment and approval by the Office of Research Compliance Macro Institutional Review Board in 2002. Surveys that fulfill the criteria set forth by the Department of Homeland Security are classified as DHS-7 Program authorized, accompanied by the requisite approval documentation. The Institutional Review Board of ICF International followed the regulations established by the US Department of Health and Human Services for the Protection of Human Subjects (45 CFR 46).

## Funding

 The authors received no specific funding for this work.

## References

[1] Ajayi AI, Mudefi E, Owolabi EO (2021). Prevalence and correlates of sexual violence among adolescent girls and young women: findings from a cross-sectional study in a South African university. BMC Womens Health.

[2] Alkan Ö, Tekmanlı HH (2021). Determination of the factors affecting sexual violence against women in Turkey: a population-based analysis. BMC Womens Health.

[3] Altunjan T (2021). The International Criminal Court and sexual violence: between aspirations and reality. German Law Journal.

[4] Wheatley L, Mastrogiovanni C, Pebole M, McKeon G, Rosenbaum S, Rees S (2024). Physical activity levels and sedentary behavior in people who have experienced gender-based violence: a systematic review. Ment Health Phys Act.

[5] Jakubowski KP, Murray V, Stokes N, Thurston RC (2021). Sexual violence and cardiovascular disease risk: a systematic review and meta-analysis. Maturitas.

[6] Molstad TD, Weinhardt JM, Jones R (2023). Sexual assault as a contributor to academic outcomes in university: a systematic review. Trauma Violence Abuse.

[7] World Health Organization (WHO). Violence Against Women Prevalence Estimates, 2018: Global, Regional and National Prevalence Estimates for Intimate Partner Violence Against Women and Global and Regional Prevalence Estimates for Non-Partner Sexual Violence Against Women. World Report on Violence and Health. Geneva: WHO; 2021.

[8] Ghana Statistical Service (GSS), ICF. Ghana Demographic and Health Survey 2022. Accra, Ghana: GSS, ICF; 2024.

[9] Alothman HM, Abdel Rahman AR, Aderibigbe SA, Ali M (2024). Risk factors associated with intimate partner violence (IPV) against Jordanian married women: a social ecological perspective. Heliyon.

[10] Prasad S, Periyan R (2019). Factors influencing intimate partner violence. Indian J Community Health.

[11] Ditsela M, Diala A, Ozoemena R (2024). African feminism and the recognition of cohabitation under customary law. Afr Dev.

[12] Obeng-Hinneh R, Kpoor A (2022). Cohabitation and its consequences in Ghana. J Fam Issues.

[13] Banarjee S (2020). Identifying factors of sexual violence against women and protection of their rights in Bangladesh. Aggress Violent Behav.

[14] Ghana Statistical Service (GSS). GDHS Key Indicators Report. Accra, Ghana: GSS; 2022.

[15] Wulandari RD, Laksono AD, Prasetyo YB, Nandini N (2022). Socioeconomic disparities in hospital utilization among female workers in Indonesia: a cross-sectional study. J Prim Care Community Health.

[16] Oyedele O, Shinedima N (2024). Determinants of intimate partner violence prevalence against mothers with under-5 children in Namibia: a cross-sectional study. SAGE Open.

[17] Aborisade RA (2021). Untold stories of violence experienced by female students in cohabitation relationship on Nigerian university campuses. Partner Abuse.

[18] Negussie YM, Seifu BL, Asnake AA, Fente BM, Melkam M, Bezie MM (2024). Sexual violence against ever-married reproductive-age women in East Africa: further analysis of recent demographic and health surveys. BMC Public Health.

[19] Badan Pusat Statistik (BPS). Prevalensi Kekerasan Terhadap Perempuan di Indonesia, Hasil SPHPN 2016. Berita Resmi Statistik; 2017.

[20] Jabbi A, Ndow B, Senghore T, Sanyang E, Kargbo JC, Bass P (2020). Prevalence and factors associated with intimate partner violence against women in the Gambia: a population-based analysis. Women Health.

[21] Fan S, Koski A (2022). The health consequences of child marriage: a systematic review of the evidence. BMC Public Health.

[22] Morzk MH, Shoaib FT, Abdel-Fatah NA (2025). Socioeconomic and demographic determinants of marital violence against women in Palestine. J Popul Soc Stud.

[23] Kebede AA, Aklil MB, Gessesse DN, Tsega NT, Temesgan WZ, Abegaz MY (2022). Nearly half of women have experienced intimate partner violence during pregnancy in Northwest Ethiopia, 2021; the role of social support and decision-making power. Front Public Health.

[24] Laksono AD, Wulandari RD, Matahari R, Suharmiati Suharmiati (2023). Socioeconomic differences of intimate partner violence among married women in Indonesia: does poverty matter?. Indian J Community Med.

[25] van Schendelstraat A, van Berlo W, Ploem R. Sexual Violence: Knowledge File. Utrecht: Rutgers; 2018.

[26] Chen XY, Lo CKM, Ho FK, Leung WC, Ip P, Chan KL (2022). Changing patterns of intimate partner violence against pregnant women: a three-year longitudinal study. Int J Environ Res Public Health.

[27] Hamzavi Zarghani N, Nazari M, Shayeghian Z, Shahmohammadi S (2016). Social support in the pregnant and non-pregnant women and its associated dimensions. J Nurs Midwifery Sci.

[28] Islam MJ, Broidy L, Mazerolle P, Baird K, Mazumder N (2021). Exploring intimate partner violence before, during, and after pregnancy in Bangladesh. J Interpers Violence.

[29] Kulhawik R, Zborowska K, Grabarek BO, Boroń D, Skrzypulec-Plinta V, Drosdzol-Cop A (2022). Changes in the sexual behavior of partners in each trimester of pregnancy in Otwock in Polish couples. Int J Environ Res Public Health.

[30] Maruyama N, Horiuchi S, Kataoka Y (2023). Prevalence and associated factors of intimate partner violence against pregnant women in urban areas of Japan: a cross-sectional study. BMC Public Health.

[31] Tessema ZT, Gebrie WM, Tesema GA, Alemneh TS, Teshale AB, Yeshaw Y (2023). Intimate partner violence and its associated factors among reproductive-age women in East Africa: a generalized mixed effect robust Poisson regression model. PLoS One.

[32] Laksono AD, Wulandari RD (2022). Violence against pregnant women in Indonesia. Iran J Public Health.

